# Cardiovascular Diseases: Therapeutic Potential of SGLT-2 Inhibitors

**DOI:** 10.3390/biomedicines11072085

**Published:** 2023-07-24

**Authors:** Weronika Frąk, Joanna Hajdys, Ewa Radzioch, Magdalena Szlagor, Ewelina Młynarska, Jacek Rysz, Beata Franczyk

**Affiliations:** Department of Nephrology, Hypertension and Family Medicine, Medical University of Lodz, ul. Żeromskiego 113, 90-549 Łódź, Poland; frweronika@gmail.com (W.F.); joanna.hajdys@gmail.com (J.H.); ewa.m.radzioch@gmail.com (E.R.); szlagor.magdalena@gmail.com (M.S.); jacek.rysz@umed.lodz.pl (J.R.); bfranczyk-skora@wp.pl (B.F.)

**Keywords:** diabetes mellitus, heart failure, sodium/glucose cotransporter 2 (SGLT2) inhibitors, bexagliflozin

## Abstract

Cardiovascular diseases (CVD) are a global health concern, affecting millions of patients worldwide and being the leading cause of global morbidity and mortality, thus creating a major public health concern. Sodium/glucose cotransporter 2 (SGLT2) inhibitors have emerged as a promising class of medications for managing CVD. Initially developed as antihyperglycemic agents for treating type 2 diabetes, these drugs have demonstrated significant cardiovascular benefits beyond glycemic control. In our paper, we discuss the role of empagliflozin, dapagliflozin, canagliflozin, ertugliflozin, and the relatively recently approved bexagliflozin, the class of SGLT-2 inhibitors, as potential therapeutic targets for cardiovascular diseases. All mentioned SGLT-2 inhibitors have demonstrated significant cardiovascular benefits and renal protection in clinical trials, in patients with or without type 2 diabetes. These novel therapeutic approaches aim to develop more effective treatments that improve patient outcomes and reduce the burden of these conditions. However, the major scientific achievements of recent years and the many new discoveries and mechanisms still require careful attention and additional studies.

## 1. Introduction

Diabetes mellitus (DM) is a metabolic disease related to chronic hyperglycemia caused by impaired insulin secretion and/or action. It occurs mainly in the older population and is often undiagnosed. More than 400 million adults worldwide suffer from it, and it is estimated that this number will increase by more than 50% in the next 20 years [1].

Persistently increased levels of glucose in the blood result in symptoms such as polyuria, polydipsia, drowsiness, or weight loss. The consequences of uncontrolled diabetes are ketoacidosis or nonketotic hyperosmolar syndrome. Chronically elevated blood glucose levels are associated with the development of numerous complications, such as nephropathy, neuropathy, and retinopathy. However, the leading causes of morbidity and mortality in both type 1 and type 2 DM are heart failure and cardiovascular disorders [2].

DM and heart failure (HF) are among the most widespread diseases in the adult population, and their numbers are increasing with age. It is very common for the two illnesses to co-exist in the same patient, and in people over the age of 65, as many as 22% of people with type 2 diabetes have HF simultaneously [2,3,4]. The co-existence of these two diseases is complex and bidirectional. The risk of developing HF is over twice as high in patients with diabetes than in those without diabetes [5]. Furthermore, it increases the hospitalization rate and worsens cardiovascular outcomes. The prognosis in this group is far worse, and mortality from all causes is enhanced, but especially from cardiovascular causes [6]. Research shows that HF is also an independent predictor of clinical prognosis, both fatal and nonfatal, in patients with DM.

Diabetic cardiomyopathy is a state of ventricular dysfunction in the absence of other cardiac risk factors in diabetics [7]. There are various mechanisms that contribute to diabetic cardiomyopathy. This includes systemic metabolic disorders, subcellular component abnormalities, numerous molecular mechanisms, or dysfunction of the renin–angiotensin–aldosterone system [8].

Most current antidiabetic drugs have an adverse effect by exacerbating cardiovascular risk factors. Some antihyperglycemic therapies, such as the use of insulin or thiazolidinediones, cause weight gain and fluid retention [9], while saxagliptin, a dipeptidylpeptidase 4 (DDP4) inhibitor, is related to an increased risk of HF in comparison to standard treatment [10,11]. New classes of agents are proven to be beneficial for cardiovascular protection. They include glucagon-like peptide-1 receptor agonists (GLP-1 RAs) and sodium/glucose cotransporter 2 (SGLT2) inhibitors. SGLT-2 inhibitors have demonstrated cardiovascular benefits in large-scale clinical trials. The possible mechanisms of these profits are being widely investigated because there is a small likelihood that they are related to improved glycemic control. According to the Empagliflozin Cardiovascular Outcome Event Trial in Type 2 Diabetes Mellitus Patients–Removing Excess Glucose (EMPA-REG OUTCOME) study, treatment with one of the SGLT-2 inhibitors, empagliflozin, decreased the rate of cardiovascular death and hospitalization for HF in diabetic patients [12]. Empagliflozin is especially recommended for patients with prevalent cardiovascular diseases (CVD) to reduce the risk of death [13]. However, the choice of drug for CVD prevention should be based on the presence of risk factors and the co-existence of CVD.

SGLT-2 inhibitors that are nowadays available in the United States are empagliflozin, dapagliflozin, canagliflozin, and ertugliflozin. In the European Union, we also have sotagliflozin. Nevertheless, on 23 January 2023, the U.S. Food and Drug Administration (FDA) approved a new antihyperglycemic agent called bexagliflozin. It has been proven to significantly improve glycemic control with a single daily dose of 20 mg. Importantly, in addition to its hypoglycemic effect, it also shows a systolic blood pressure-lowering effect [14].

The aim of this review is to list, discuss, and compare each individual SGLT-2 inhibitor, with a focus on the recently approved bexagliflozin. We also want to outline the most important properties and side effects of this particular group of medications.

## 2. Empagliflozin

The SGLT-2 inhibitors, which include empagliflozin, are one of the more recent groups used in antihyperglycemic therapy. On the basis of numerous studies and comparative meta-analyses, it was noted that it significantly reduced HF hospitalizations in patients with both stable cardiovascular disease and acute HF. Empagliflozin reduced all-cause and cardiovascular mortality and reduced the risk of cardiovascular disease, regardless of the initial risk [6,15,16,17]. It also significantly improved myocardial function in patients, regardless of their ejection fraction levels [18,19,20,21]. The mechanisms by which SGLT-2 inhibitors both improve glycemic levels and improve parameters in cardiovascular disease are not fully understood. However, ongoing studies have noted that the use of empagliflozin reduces interstitial fibrosis of the ventricular myocardium, improves aortic stiffness, and induces anti-inflammatory effects [22,23,24,25,26]. As with other SGLT-2 inhibitors, it also reduces the amount of pericardial fat [27]. In addition, improvements in hematocrit and hemoglobin were found in patients, which, like the aforementioned findings, may have contributed to a reduction in HF hospitalizations and mortality in patients both with and without diabetes [18,19,22,28,29].

Preclinical studies report that the use of empagliflozin reverses the effect of glucotoxicity by lowering serum methylglyoxal levels and attenuating AGE/RAGE signaling [30,31]. It was also observed to inhibit NADPH oxidase and reduce reactive oxygen species (ROS) production, leading to a reduction in oxidative stress on endothelial cells. In addition, it led to increased NO production by improving endothelial nitric oxide synthase (eNOS) activity [30,32]. Empagliflozin was also responsible for decreasing the expression of inflammatory molecules such as adhesion molecules (ICAM-1, VCAM-1) and macrophage markers (MCP-1), contributing to the reduced induction of endothelial damage [32]. Empagliflozin also improved endothelial cell health by maintaining the integrity of the glycocalyx [33]. Moreover, the cardioprotective and anti-inflammatory effects of empagliflozin included a reduction in the concentrations of eicosanoids such as PGE2 and TXB2, which led to vascular wall damage and vessel lumen constriction [34]. Notably, empagliflozin contributed to the decline of atherosclerotic plaque by reducing the levels of circulating TNF alpha, IL-6, and MCP-1 in the blood [35].

The first study to evaluate the effect of SGLT-2 inhibitors on cardiovascular events was EMPA-REG OUTCOME [12]. The study was a randomized, double-blind, placebo-controlled trial, and its principal objective was to evaluate the effect of empagliflozin on the occurrence of cardiovascular events in adults with type 2 diabetes and established cardiovascular disease. The primary outcome was the occurrence of one of the events, such as death from cardiovascular causes, nonfatal myocardial infarction, or nonfatal stroke, while the key secondary outcome was the primary outcome along with hospitalization for unstable angina. After a mean follow-up of 3.1 years, it was noted that patients receiving empagliflozin had a significantly lower incidence of the primary outcome compared to the placebo group (10.5% and 12.1%, respectively). In contrast, given the similar incidence of hospitalization for unstable angina in both groups, there was no meaningful difference in the key secondary outcome. Furthermore, it was observed that empagliflozin markedly reduced cardiovascular mortality, any cause mortality, and hospitalization for HF [12]. During follow-up, a slight decrease in weight, systolic and diastolic blood pressure without an increase in heart rate, and elevated hematocrit and hemoglobin values were noted in patients. In a post-analysis of the EMPA-REG OUTCOME trial, it was concluded that an increase in hematocrit and hemoglobin levels was associated with a decreased risk of HF hospitalization and death from HF. This was related to enhanced myocardial function, improved oxygen supply, and reduced cardiac preload and afterload [36].

In the randomized EMPEROR-Reduced trial, 3730 patients with chronic HF (NYHA 2–4) and a left ventricular ejection fraction (LVEF) of 40% or less were screened to assess the effect of empagliflozin on the incidence of cardiovascular death or first HF hospitalization (the first outcome), as well as the rate of all HF hospitalizations (the first-second outcome) [37]. The primary composite outcome appeared in a distinct minority of those subjects who took empagliflozin in comparison to those who took a placebo, 19.4% and 24.7% (HR = 0.75, *p* < 0.001), respectively. The preceding effect was similar in both the diabetic and non-diabetic groups (hazard ratios of 0.72 and 0.78 in comparison with the placebo group, respectively). During the course of the trial, the number of all hospitalizations for HF in patients taking empagliflozin was lower as compared to the placebo group (HR = 0.70, *p* < 0.001). It should also be emphasized that patients experienced significant improvements in cardiovascular and renal outcomes regardless of their baseline diabetes status [21].

A subsequent study was EMPEROR-Preserved [20], enrolling patients with chronic HF (NYHA 2–4) and LVEF above 40%. Both the first outcome and secondary outcomes were similar to those of the EMPEROR-Reduced trial. The onset of the first outcome was lower in the empagliflozin group (13.8%) than in the placebo group (17.1%). Both hospitalizations for HF and deaths from cardiovascular causes decreased. Importantly, the observed changes were the same across subgroups, including patients with diabetes (16.3% vs. 19.8%, HR = 0.79) and without diabetes (11.5% vs. 14.5%, HR = 0.78), according to the placebo group. In the first-second outcome, the rate of hospitalization from HF was also lower in the empagliflozin group than in the placebo group (HR = 0.73, *p* < 0.001), and the time to first hospitalization was prolonged. In the post-analysis of the study, improvements in parameters were observed in the form of a decrease in glycated hemoglobin levels and body weight and an increase in hemoglobin levels [38]. In addition, a decrease in NT-proBNP levels was noted, which was initially similar in patients with and without diabetes. However, in the following weeks, a more pronounced decline was noted in patients with diabetes.

The EMPA-Tropism trial examined whether empagliflozin also had a positive effect on heart failure with reduced ejection fraction (HFrEF), exercise capacity, and quality of life in non-diabetic patients [22]. The first outcome was to determine whether empagliflozin attenuates adverse myocardial remodeling as assessed by improvements in left ventricular end-diastolic volume (LVEDV) and left ventricular end-systolic volume (LVESV). In the second outcome, among others, changes in peak oxygen consumption, left ventricular mass, LVEF, distance in the 6 min walk test (6MWT), and quality of life were assessed using the KCCQ-12 scale. After a 6-month follow-up, substantial improvement was noted in LVEDV in the empagliflozin versus placebo group (−25.1 ± 26.0 mL vs. −1.5 ± 25.4 mL; *p* < 0.001) compared to the beginning of the study [39]. Similar changes were observed for left ventricular end-systolic diameter (LVESD) (−26.6 ± 20.5 mL vs. −0.5 ± 21.9 mL, *p* < 0.001). In addition, the empagliflozin group had a significant reduction in left ventricular mass (−17.8 ± 31.9 g vs. 4.1 ± 13.4 g; *p* < 0.001) and a more pronounced increase in LVEF (6 ± 4.2 vs. −0.1 ± 3.9; *p* < 0.001) in comparison with placebo. There was a notable enhancement in peak oxygen consumption (1.1 ± 2.6 mL/min/kg) and distance extension in the 6MWT (81 ± 64 m) in the empagliflozin group [22,39]. All the above-mentioned elements contributed to a meaningful improvement in the patient’s quality of life.

EMPULSE was a prominent trial that investigated empagliflozin’s effects in people with acute HF [40]. The trial aimed to introduce empagliflozin into HF treatment while patients were still in the hospital. The developments observed during the trial provided clinically significant benefits to patients at the same time as providing no safety concerns about its use [41]. The main objective was to improve survival, reduce symptoms, and reduce the incidence of heart failure events. There were 11 deaths in the empagliflozin group (4.2%), while 22 patients (8.3%) died in the placebo group. Sixty-seven patients had at least one heart failure event (HFE) during the study, with twenty-eight patients in the empagliflozin group and thirty-nine in the placebo group (10.6% and 14.7%, respectively). Furthermore, there was a greater absolute change in the Kansas City Cardiomyopathy Questionnaire Total Symptom Score (KCCQ-TSS) from baseline to day 90 in patients in the empagliflozin group (HR = 4.45; 95% CI 0.32–8.59) and a significant reduction in NT-proBNP levels (HR = 0.90, 95% CI 0.82–0.98) [41].

The individual SGLT-2 inhibitors have a similar range of action; therefore, an important factor comparing them to one another will be cardiovascular events and the occurrence of adverse events [42]. In a meta-analysis conducted by Zelniker et al. [16] both empagliflozin, canagliflozin, and dapagliflozin were associated with reduced hospitalizations for HF and reduced progression of kidney disease. However, in patients with atherosclerotic CVD, the positive effect of empagliflozin on reducing cardiovascular death was more pronounced than with the other SGLT-2 inhibitors. A similar effect was observed for all-cause mortality [43,44]. In the retrospective trial conducted by Suzuki et al. [45], the incidence of subsequent cardiovascular risk in terms of HF, myocardial infarction, angina, stroke, and atrial fibrillation was compared in accordance with individual SGLT-2 inhibitors. It turned out that no significant differences in the risk of the above-mentioned cardiovascular events were observed between empagliflozin, dapagliflozin, canagliflozin, or other SGLT-2 inhibitors (ipragliflozin, tofogliflozin, and luseogliflozin). A comparable effect was observed in reducing HF progression [43]. Entirely different conclusions were reached in a study by Jing et al. [44], in which empagliflozin was associated with a more favorable effect on the occurrence of cardiovascular events than canagliflozin or dapagliflozin. Additionally, according to Tang et al. [46], it was more likely to reduce the risk of HF or HF requiring hospitalization compared to the other SGLT-2 inhibitors. The studies comparing empagliflozin with other SGLT-2 inhibitors are summarized in Table 1.

## 3. Dapagliflozin

**Figure 1:**
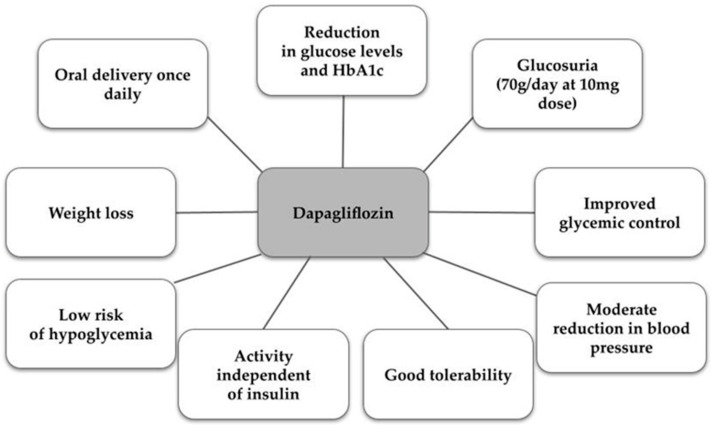
Characteristics of dapagliflozin [50,52].

One example of a selective SGLT2 inhibitor is dapagliflozin, used under the trade name Forxiga^®^ in Europe or Farxiga^®^ in the US, in doses of 5 or 10 mg. It was approved in 2012 by the European Medicines Agency (EMA) and in 2014 by the Food and Drug Administration (FDA) [47,48]. As for the drug’s pharmacokinetics, due to its approximately 14 h half-life, it can be used once daily, reaching maximum plasma concentrations after about 2 h, while its metabolites are excreted mainly in the urine and feces [47,48,49]. Dapagliflozin acts mainly in the proximal tubule of the kidney, and the mechanism involves reducing the reabsorption of glucose. This increases the excretion of glucose in the urine, which leads to the desired hypoglycemic effect. Indirectly, there is a partial reduction in body weight through negative energy balance and a reduction in blood pressure due to osmotic diuretic action—mild natriuresis [50,51]. The characteristics of the properties and actions of dapagliflozin are presented in Figure 1 [50,52].

In the European Union, it is used in monotherapy or combination therapy in T2DM when patients fail to achieve normal glycemic control despite lifestyle changes, i.e., diet and adequate exercise. Dapagliflozin has been shown in numerous studies to reduce hospital admissions for heart failure and the rate of death from cardiovascular causes in both patients with and without T2DM [49,53]. In patients with parenchymal CVD, it probably caused a reduction in renal disease progression [49]. Dapagliflozin also has a partial metabolic effect caused by increasing muscle insulin sensitivity [54]. Worthy of mention is the international, randomized Dapagliflozin Effect on Cardiovascular Events–Thrombolysis in Myocardial Infarction 58 (DECLARE–TIMI 58) study. It had a double-blind, placebo-controlled, phase 3 trial to evaluate the effect of dapagliflozin in patients with T2DM and established atherosclerotic cardiovascular disease or multiple atherosclerotic cardiovascular risk factors on cardiovascular events [1]. Positive effects on the kidneys, such as natriuresis and improved endothelial function, have also been observed [49]. The study included 17,160 patients diagnosed with T2DM. It showed that the use of dapagliflozin resulted in reduced cardiovascular deaths and hospitalizations for HF, regardless of ejection fraction [48,55,56]. In contrast, SGLT2 treatment did not lead to a statistically significant reduction in MACE [48,57,58]. Another study, Dapagliflozin and Prevention of Adverse Outcomes in Heart Failure (DAPA-HF), included 4744 patients with heart failure with or without T2DM, reduced EF (≤40%), NYHA score II–IV, and elevated NT-proBNP. Patients were randomly assigned to take one 10 mg tablet of dapagliflozin daily. Less than 42% of the subjects had additional T2DM [59,60]. The characteristics of patients with T2DM participating in the DAPA-HF study are presented in Table 2.

These studies indicated that HF and HFrEF, regardless of the presence or absence of T2DM, who were consuming dapagliflozin, had a lower risk of cardiovascular death [60]. An important cardioprotective component of dapagliflozin is the reduction in cardiac preload and afterload through blood volume reduction caused by mild diuresis. Reducing oxidative stress in cells may improve the structure of damaged cardiac cells, which consequently improves long-term prognosis [56]. Studies in mice have shown a vasodilatory effect of dapagliflozin on the thoracic aorta, depending on the voltage of potassium channels [30]. This suggested a direct effect on vascular cells for both acute and chronic treatment. The vascular response resulted in a reduction in oxidative stress by reducing glycation [30,61]. The beneficial effect on the endothelium is due to several vasodilatory mechanisms, such as reduced infiltration of macrophages into the myocardium and activation of eNOS phosphorylation [61].

As for contraindications to the use of the drug, these are, of course, a history of hypersensitivity reactions, for example, angioedema or anaphylactic reaction, and patients on dialysis therapy [62]. Adverse reactions during dapagliflozin pharmacotherapy did occur, but they represented a small percentage.

Patients could experience rhinosinusitis, upper respiratory tract infections in general, headaches, back pain, or the occurrence of diarrhea [49]. The most well-known complication due to glucosuria is emerging urinary tract infections, including cases of urosepsis or pyelonephritis. There is also a risk of fungal genital infections or life-threatening Fournier gangrene [1,57,59]. The use of SGLT2 inhibitors has been linked to the occurrence of both hypoglycemia and cases of diabetic ketoacidosis, which have also led to deaths. Of course, these cases occurred only in patients with T2DM [1,49,58]. There were also transient decreases in renal creatinine clearance, and some patients presented clinical signs of hypotension [49,62]. When thinking about dapagliflozin, it is worth remembering a number of its systemic effects. In addition to its obvious and best-studied hypoglycemic and glycated hemoglobin-reducing effects, it also has cardioprotective and renoprotective properties [49]. The drug’s mechanism of action also determines a positive effect on metabolic syndrome, which will indirectly contribute to the reduction of cardiovascular events in these patients [63]. What is important is that it is well tolerated by a wide range of patients, regardless of a history of CVD [49].

## 4. Canagliflozin

Canagliflozin is one of the SGLT2 inhibitors. Primarily used to treat type 2 diabetes, it has also been studied for its potential cardiovascular benefits. Principally, canagliflozin reduced the risk of cardiovascular events in people with type 2 diabetes, regardless of the coincidence of CVD [64,65,66]. Canagliflozin improves several cardiovascular risk factors, including lowering body weight and blood pressure, body composition, uric acid levels, vascular stiffness, pulse pressure, cardiac workload, and magnesium levels [67,68,69,70]. A growing body of literature points to the significant role of SGLT2 inhibitors in improving symptoms in patients with HF [71]. Canagliflozin improves patients’ symptom burden, driven primarily by volume and hemodynamic effects. The protection provided may be a result of natriuresis-induced decreases in preload and afterload [72], systemic blood pressure lowering [73,74], modification of the intrarenal renin–angiotensin axis [75], and reduction in arterial stiffness [76]. In addition, it has shown beneficial solid effects on decreasing cardiovascular death rates and hospitalized HF, especially in those with a history of CVD [77,78,79]. Furthermore, according to this study, canagliflozin might reduce the progression of atherosclerosis, adhesion molecules, and markers of inflammation (i.e., vascular cell adhesion molecule-1 and monocyte chemotaxis protein-1). Additionally, canagliflozin enhances atherosclerotic plaque stability in mouse models [80]. Nevertheless, the characterization of cardiac function that would identify the patient groups that would benefit from the administration of canagliflozin has not been fully investigated, despite increasing proof of its positive effects on HF.

Canagliflozin demonstrates cardioprotective benefits independent of a glucose-lowering effect, including preservation of cardiac function during myocardial ischemia. Canagliflozin considerably attenuates the size of myocardial infarcts [81,82]. Sabe et al. found that canagliflozin therapy enhances myocardial function and perfusion to the ischemic region in a swine model of chronic myocardial ischemia. These outcomes may be mediated by antioxidant signaling, adenosine monophosphate-activated protein kinase activation, and attenuation of fibrosis via decreased Jak/STAT signaling [83]. Furthermore, according to this research, the intravenous administration of canagliflozin decreased the expression of apoptotic and nitro-oxidative stress markers while increasing the phosphorylation of cardioprotective signaling mediators, such as adenosine monophosphate-activated protein kinase, acetyl-CoA carboxylase, endothelial nitric-oxide synthase, and Akt, in non-diabetic rats. Additionally, canagliflozin has been linked to a slower increase in biomarkers of cardiac wall stress, such as high-sensitivity troponin I and NT-proBNP, as well as a rise in hematocrit [84]. Correspondingly, canagliflozin inhibited the onset of systolic and diastolic dysfunction after ischemia-reperfusion damage [85]. Table 3 provides a summary of canagliflozin’s cardioprotective effects.

A remarkable cardioprotective effect against cardiac arrest and resuscitation-induced cardiac dysfunction was obtained by canagliflozin [85,86]. Interestingly, in comparison to control mice, animals pretreated with canagliflozin had better survival rates (*p* < 0.05), a faster return of spontaneous circulation (*p* < 0.01), and increased neurological scores (*p* < 0.01 or *p* < 0.001) following resuscitation. Canagliflozin may exert its effects through the STAT-3-dependent cell-survival signaling pathway, according to this study [85].

The possible benefit of canagliflozin use in the development and progression of atrial fibrillation (AF) has been suggested [87]. This study has demonstrated that the administration of canagliflozin reduces atrial electrical and structural remodeling, interstitial fibrosis, and oxidative stress levels in canine models [88]. On the contrary, this meta-analysis by Li et al. showed that SGLT2 inhibitor use is linked to a 19.33% lower risk of serious adverse events of AF and atrial flutter (AFL) when compared with placebo. However, only dapagliflozin (1.02% vs. 1.49%; RR 0.73; 95% CI 0.59–0.89; *p* = 0.002; I^2^ 0%), but not canagliflozin (1.00% vs. 1.08%; RR 0.83; 95% CI 0.62–1.12; *p* = 0.23; I^2^ 0%), significantly reduced AF and AFL. Further studies are required to establish whether canagliflozin similarly exerts protective effects against AF/AFL development [89]. A summary of canagliflozin’s effects on the cardiovascular system is shown in Table 4.

## 5. Ertugliflozin

Ertugliflozin is an SGLT2 inhibitor that is used as an adjunct therapy for the treatment of DM. The cardioprotective effects of ertugliflozin among individuals with CVD have not been extensively investigated in clinical trials.

In preclinical models, ertugliflozin has been found to improve cardiac energy metabolism by increasing the availability of ketone bodies as an alternative energy source for the heart. This shift in substrate utilization may help preserve cardiac function in conditions such as HF. Furthermore, the drug has demonstrated the ability to attenuate cardiac remodeling, including left ventricular hypertrophy, fibrosis, and inflammation, pathological changes commonly observed in CVD [90,91].

The VERTIS CV trial assessed the impact of ertugliflozin in patients with type 2 diabetes and CVD, including those with a history of HF and known a pre-trial ejection fraction. The study demonstrated that treatment with ertugliflozin reduced the occurrence and total hospitalizations for HF events. This benefit was observed in patients with and without a history of HF, as well as in those with reduced or preserved ejection fraction [92].

The trial also revealed that the risk reduction for the first hospitalization for HF with ertugliflozin was consistent across most baseline subgroups. However, a greater benefit was observed in three specific populations: those with an estimated glomerular filtration rate (eGFR) less than 60 mL/min/1.73 m^2^, albuminuria, and diuretic use. Additionally, ertugliflozin use was associated with decreased albuminuria and preservation of eGFR over time, indicating its potential for kidney protection in patients with type 2 diabetes and CVD [93,94].

Overall, these findings suggest that ertugliflozin may have additional cardiovascular positive effects, apart from its effects on lowering glucose levels. Thus, it might be a promising therapeutic method for those with HF and CVD. However, further research is needed to fully comprehend the underlying mechanisms responsible for these effects.

## 6. Bexagliflozin

Bexagliflozin is a novel agent approved by the FDA in 2023. This highly potent and selective inhibitor of SGLT2 is indicated for adults with type 2 DM with an eGFR greater than 30 mL/min/1.73 m^2^. It is available as 20 mg oral tablets, recommended to be taken once daily, regardless of the meal. Patients with diabetes and mild to moderate kidney failure have fewer treatment options compared to those with preserved kidney function. Dosage modifications have been presented in Table 5 and Table 6.

A 96-week phase 2 clinical study showed that bexagliflozin monotherapy led to a long-lasting, clinically relevant improvement in glycemic control, with a significant reduction in weight and blood pressure [14]. In a clinical study of patients with T2DM and co-existing chronic kidney disease (CKD) (at stage 3a/3b), bexagliflozin was well tolerated and demonstrated a decrease in hemoglobin A1c levels as well as body weight, systolic blood pressure, and albuminuria [95]. Allegretti et al. [95] have also revealed adverse events such as urinary tract infections and genital mycotic infections. However, those findings have been previously attributed to SGLT2 inhibition. A summary of side effects is shown in Table 7.

Bexagliflozin has been proven to be non-inferior to other SGLT2 inhibitors. Halvorsen et al. [97] revealed that its effects on body weight and blood pressure were even superior to commonly prescribed add-on therapy with the DPP-4 inhibitor, sitagliptin. It has also been non-inferior to glimepiride in lowering HbA1c [98]. Furthermore, it has achieved superiority over glimepiride in the reduction of body mass and systolic blood pressure (SBP) [5]. Another important finding was the demonstration of remarkably fewer hypoglycemic events than with glimepiride. Importantly, McMurray et al. [99] have shown bexagliflozin’s non-inferiority for hard clinical outcomes in high-risk CVD cohorts.

## 7. SGLT-2 Inhibitors’ Effects on the Kidney and Heart

**Figure 2:**
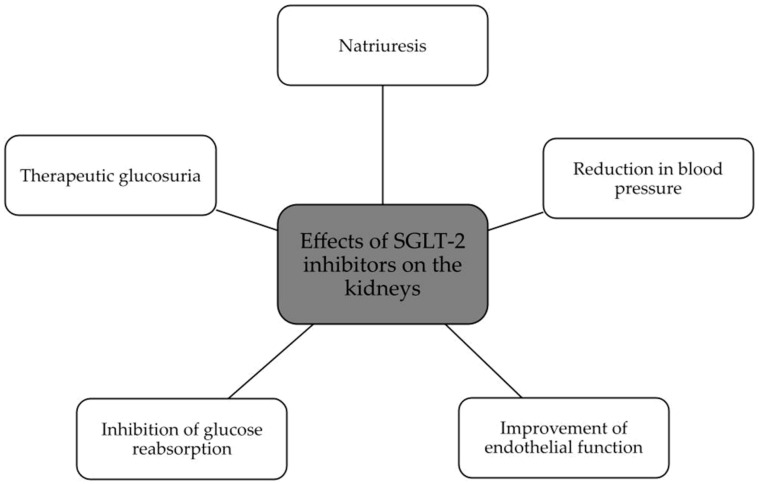
Beneficial effects of SGLT2 inhibitors on the kidney [47,49,51].

**Figure 3:**
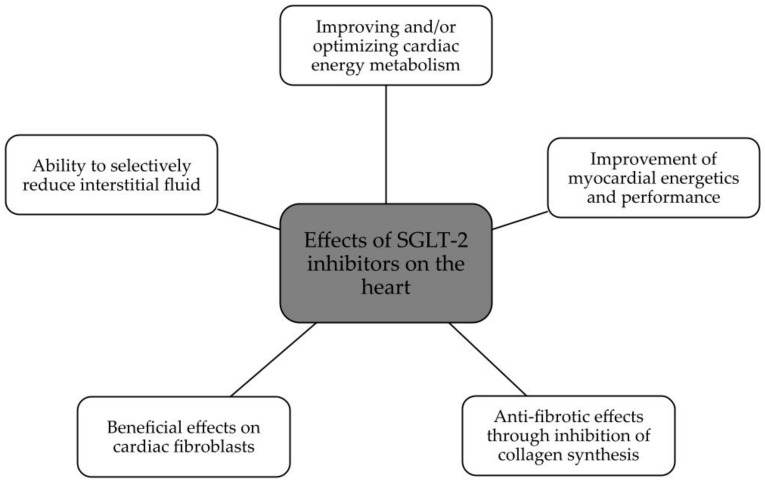
Beneficial effects of SGLT2 on the heart [26].

The summary of SGLT2 inhibitors’ effects on the kidney and heart is presented in Figure 2 and Figure 3.

## 8. Conclusions

According to recent epidemiological data [100], type 2 DM is closely related to cardiovascular disease development. Heart failure, as a leading cause of morbidity and mortality in diabetics, is especially perilous among this group of patients [101]. Since 2008, the FDA has required proof of cardiovascular safety for new glucose-lowering therapies [102]. SGLT2 inhibitors have revealed a number of cardio-protective beneficial effects in both primary and secondary prevention [103]. Not only do they reduce cardiovascular events, improve HF symptoms, or decrease cardiovascular death rates and hospitalizations for HF, but they are also proven to preserve cardiac function during myocardial ischemia as well as slow the progression of AF. Evidence of the clinical benefits of this new antihyperglycemic therapy has led to a relevant change in the care paradigm across several high-risk populations.

Beyond their glucose-lowering effects, all of the mentioned SGLT2 inhibitors have been proven to have additional cardiovascular benefits and could be a promising treatment option for patients with CVD and HF. Empagliflozin has been proven to be the most effective SGLT2 inhibitor in lowering the risk of HF. It is also superior in reducing death from cardiovascular causes to canagliflozin or dapagliflozin. Undeniably, bexagliflozin has been actively awaited, mainly due to the ever-increasing prevalence of T2DM as well as increased morbidity and mortality from associated cardiovascular consequences. Studies have shown its non-inferiority for hard clinical outcomes in high-risk CVD cohorts [99]. Nowadays, it is also undergoing clinical development for the treatment of essential hypertension in the USA.

In conclusion, it has been proven that patients with HF and DM may benefit from SGLT2 inhibitors. The balance of profits and adverse impacts depends on the individual risk profiles. 

## Figures and Tables

**Table 1:** A comparison of empagliflozin with other SGLT-2 inhibitors in patients with type 2 diabetes.

Study	Tang et al. [46]	Zelniker et al. [16]	Täger et al. [43]	Suzuki et al. [45]	Jiang et al. [44]
**Year**	2016	2019	2021	2022	2022
**Study design**	Meta-analysis	Meta-analysis	Meta-analysis	Retrospective cohort study	Meta-analysis
**No of patients**	28,859	34,322	74,874	25,315	70,574
**Patient’s characteristics**	Patients > 18 years old with T2DM	Mean age 63.5 years, 60.2% patients with atherosclerotic CVD, 11.3% patients with history of HF, 14.9% patients with eGFR < 60 mL/min per 1.73 m^2^	Patients 52–69 years old, HbA1c level between 7.2% and 9.3%	Median age 52 years, median HbA1c level 7.5%	Mean age 59.2 years old, mean HbA1c level 8.3%
**SGLT2 inhibitors**	Canagliflozin, dapagliflozin, or empagliflozin vs. placebo or other active anti-diabetic treatments	Empagliflozin 10 mg, 25 mg, canagliflozin 100 mg, 300 mg, dapagliflozin 10 mg	Canagliflozin, dapagliflozin, empagliflozin, ertugliflozin	Empagliflozin, dapagliflozin, canagliflozin, other SGLT2 inhibitors (ipragliflozin, tofogliflozin, luseogliflozin)	Empagliflozin 5 mg, 10 mg, 25 mg, 50 mg, canagliflozin 100 mg, 300 mg, dapagliflozin 2.5 mg, 5 mg, 10 mg, placebo
**Comparison of SGLT2 inhibitors**	Empagliflozin significantly lower the risk of MACE and any-cause mortality than placebo and other SGLT2 inhibitors. Furthermore, empagliflozin lower risk of HF and HF requiring hospitalization.	Empagliflozin has superior effect on reducing death from cardiovascular causes than canagliflozin or dapagliflozin. There is an increased risk of fractures and amputations with canagliflozin.	Empagliflozin is superior to canagliflozin and dapagliflozin in reducing all-cause mortality and cardiovascular mortality. However, all without significant differences reduce HF worsening.	There were no relevant differences in the risk of myocardial infarction, angina pectoris, heart failure, atrial fibrillation and stroke among individual SGLT2 inhibitors.	Empagliflozin is associated with significantly lower risk of all-cause mortality and cardiovascular events than canagliflozin and dapagliflozin.

T2DM, type 2 diabetes mellitus; MACE, major adverse cardiovascular events; HF, heart failure; CVD, cardiovascular disease; eGFR, estimated glomerular filtration rate.

**Table 2:** Characteristics of patients with T2DM participating in the DAPA-HF study [60].

Characteristics in Patients with T2DM
Higher body mass index
More obese individuals
More people with a history of myocardial infarction, ischemic disease, coronary artery disease
NYHA score II–IV
Higher serum NT-proBNP levels
Lower values of mean eGFR
More patients with hypertension

NYHA, New York Heart Association; eGFR, estimated glomerular filtration rate.

**Table 3:** Cardioprotective effects of canagliflozin on myocardial infarction.

Study	Year	Study Design	Participants	Findings
Januzzi, J.L.; Butler, J.; Jarolim, P. et al. [83]	2017	Randomized, double-blind, placebo-controlled	666 patients with DM type 2 and high cardiovascular risk	Canagliflozin had a favorable effect on cardiovascular biomarkers in older adults with DM type 2. In comparison to a placebo, the administration of canagliflozin in older patients with DM type 2 resulted in a significant delay in the increase of serum NT-proBNP and hsTnI levels
Huynh, K. [81]	2017	Randomized, controlled	10,142 patients with type 2 DM and high cardiovascular risk	Canagliflozin was associated with a lower risk of cardiovascular events in patients with DM type 2.
Lim, V.G.; Bell, R.M.; Arjun, S.; Kolatsi-Joannou, M.; Long, D.A.; Yellon, D.M. [80]	2019	Randomized, double-blind, placebo-controlled	Diabetic and non-diabetic rats	Canagliflozin attenuated myocardial infarction in both diabetic and non-diabetic mice. The observed effects were independent of glucose levels during the occurrence of ischemia/reperfusion injury.
Sayour, A.A.; Korkmaz-Icöz, S.; Loganathan, S. et al. [84]	2019	Randomized, controlled	Non-diabetic male rats	Acute canagliflozin treatment protected against in vivo myocardial ischemia-reperfusion injury in non-diabetic male rats and enhanced endothelium-dependent vasorelaxation.
Sabe, S.A.; Xu, C.M.; Sabra, M.; et al. [82]	2023	Randomized, controlled	Swine model of chronic myocardial ischemia	Canagliflozin improved myocardial perfusion, fibrosis, and function in a swine model of chronic myocardial ischemia.

DM, diabetes mellitus; NT-proBNP, N-terminal pro-B-type natriuretic peptide; hsTnI, high-sensitivity troponin I.

**Table 4:** Canagliflozin effects on cardiovascular system.

Cardiovascular Benefit	Effect on Cardiovascular Risk Factors	Mechanism of Action
Reduction in cardiovascular events	Lowered body weight and blood pressure, improved body composition, uric acid levels, vascular stiffness, pulse pressure, cardiac workload, and magnesium levels.	Not fully investigated.
Improvement in heart failure symptoms	Improvement in volume and hemodynamic effects, natriuresis-induced decreases in preload and afterload, systemic blood pressure lowering, modification of the intrarenal renin–angiotensin axis, and reduction in arterial stiffness.	Not fully investigated.
Reduction in cardiovascular death rates and hospitalization for heart failure	Decrease in atherosclerosis progression, adhesion molecules, and markers of inflammation.	Enhanced atherosclerotic plaque stability in mouse models.
Preservation of cardiac function during myocardial ischemia	Attenuation of myocardial infarct size.	Antioxidant signaling, adenosine monophosphate-activated protein kinase activation, and attenuation of fibrosis via decreased Jak/STAT signaling.
Slower increase in biomarkers of cardiac wall stress	Inhibition of onset of systolic and diastolic dysfunction after ischemia-reperfusion damage.	Not fully investigated.
Cardioprotective effect against cardiac arrest and resuscitation-induced cardiac dysfunction	Improved survival rates, shorter return of spontaneous circulation, and higher neurological scores following resuscitation.	STAT-3-dependent cell-survival signaling pathway.
Possible benefit in the development and progression of atrial fibrillation	Reduced atrial electrical and structural remodelling, interstitial fibrosis, and oxidative stress levels in canine models.	Not fully established; conflicting results in clinical studies.

**Table 5:** Dosage modifications of bexagliflozin in renal impairment.

Renal Impairment	eGRF (mL/min/1.73 m^2^)	Dosage Modifications
Mild-to-moderate	0–89	No dosage adjustment required.
Severe	<30	Not recommended owing to the decline of glucose-lowering effect and reduction in urine output.
Dialysis	-	Contraindicated.

eGFR, estimated glomerular filtration rate.

**Table 6:** Dosage modifications of bexagliflozin in hepatic impairment.

Hepatic Impairment	Child–Pugh Score	Dosage Modifications
Mild-to-moderate	A or B	No dosage adjustment required.
Severe	C	Not studied.

**Table 7:** Side effects of bexagliflozin [96].

Side Effects	Prevention
Ketoacidosis	Consideration of predisposing factors, discontinuing bexagliflozin for at least 3 days prior to surgery and clinical situations known to predispose to ketoacidosis.
Lower limb amputation	Consideration of predisposing factors, monitoring for signs and symptoms of infection, new pain or tenderness, sores or ulcers involving the lower limbs.
Volume depletion	Assessment of volume status and renal function, monitoring for signs and symptoms of volume depletion.
Urosepsis and pyelonephritis	Evaluation patients for signs and symptoms of urinary tract infections.
Hypoglycemia with concomitant use with insulin and insulin secretagogues	lower dose of insulin or insulin secretagogue.
Necrotizing fasciitis of the perineum (Fournier’s gangrene)	Evaluation of patients for pain or tenderness, erythema, or swelling in the genital or perineal areas, along with fever or malaise.
Genital mycotic infections	Monitoring patients with a history of genital mycotic infections and those who are uncircumcised.

## Data Availability

The data used in this article are sourced from materials mentioned in the References section.

## Abbreviations

6MWT	6 min walk test
AF	Atrial fibrillation
AFL	Atrial flutter
CAD	Coronary artery disease
CKD	Chronic kidney disease
CVD	Cardiovascular disease
DAPA-HF	Dapagliflozin and Prevention of Adverse Outcomes in Heart Failure
DECLARE–TIMI 58	Dapagliflozin Effect on Cardiovascular Events–Thrombolysis in Myocardial Infarction 58
DM	Diabetes mellitus
DDP4	Dipeptidylpeptidase 4
eGFR	Estimated glomerular filtration rate
EMA	European Medicines Agency
EMPA-REG OUTCOME	Empagliflozin Cardiovascular Outcome Event Trial in Type 2 Diabetes Mellitus Patients–Removing Excess Glucose
FDA	Food and Drug Administration
GLP-1 RAs	Glucagon-like peptide-1 receptor agonists
HF	Heart failure
HFE	Heart failure event
HFrEF	Heart failure with reduced ejection fraction
LVEDV	Left ventricular end-diastolic volume
LVEF	Left ventricular ejection fraction
LVESD	Left ventricular end-systolic diameter
LVESV	Left ventricular end-systolic volume
MACE	Major adverse cardiovascular events
NYHA	New York Heart Association
SBP	Systolic blood pressure
SGLT2	Sodium/glucose cotransporter 2
T2DM	Type 2 diabetes mellitus
